# A genome-wide map of DNA replication at single-molecule resolution in the malaria parasite *Plasmodium falciparum*

**DOI:** 10.1093/nar/gkad093

**Published:** 2023-02-20

**Authors:** Francis Isidore Garcia Totañes, Jonas Gockel, Sarah E Chapman, Richárd Bártfai, Michael A Boemo, Catherine J Merrick

**Affiliations:** Department of Pathology, University of Cambridge, Tennis Court Road, Cambridge CB2 1QP, UK; Department of Molecular Biology, Radboud University, Geert Grooteplein 26-28, 6525 GA Nijmegen, The Netherlands; Department of Pathology, University of Cambridge, Tennis Court Road, Cambridge CB2 1QP, UK; Department of Molecular Biology, Radboud University, Geert Grooteplein 26-28, 6525 GA Nijmegen, The Netherlands; Department of Pathology, University of Cambridge, Tennis Court Road, Cambridge CB2 1QP, UK; Department of Pathology, University of Cambridge, Tennis Court Road, Cambridge CB2 1QP, UK

## Abstract

The malaria parasite *Plasmodium falciparum* replicates via schizogony: an unusual type of cell cycle involving asynchronous replication of multiple nuclei within the same cytoplasm. Here, we present the first comprehensive study of DNA replication origin specification and activation during *Plasmodium* schizogony. Potential replication origins were abundant, with ORC1-binding sites detected every ∼800 bp. In this extremely A/T-biased genome, the sites were biased towards areas of higher G/C content, and contained no specific sequence motif. Origin activation was then measured at single-molecule resolution using newly developed DNAscent technology: a powerful method of detecting replication fork movement via base analogues in DNA sequenced on the Oxford Nanopore platform. Unusually, origins were preferentially activated in areas of low transcriptional activity, and replication forks also moved fastest through lowly transcribed genes. This contrasts with the way that origin activation is organised in other systems, such as human cells, and suggests that *P. falciparum* has evolved its S-phase specifically to minimise conflicts between transcription and origin firing. This may be particularly important to maximise the efficiency and accuracy of schizogony, with its multiple rounds of DNA replication and its absence of canonical cell-cycle checkpoints.

## INTRODUCTION

Malaria-causing *Plasmodium* parasites cause morbidity and mortality in hundreds of millions of people every year. Severe and fatal malaria is principally caused by the species *Plasmodium falciparum*, which is responsible for ∼600 000 deaths per year (*World malaria report 2021*, World Health Organization). The extent to which these parasites can replicate in human hosts is very clinically relevant because high parasitaemia is one of the strongest predictors of severe malarial disease (1,2). Accordingly, it is important to understand the cellular and molecular biology of *Plasmodium* replication, which is fundamentally different from that of most other pathogens. Most cells—from bacterial to fungal to protozoal pathogens—replicate via canonical binary fission, whereas these early-diverging protozoans replicate primarily via schizogony. This unique process involves asynchronous replication of multiple nuclei within the same cytoplasm, prior to a single cytokinesis event producing many—and not necessarily 2^*n*^—daughter cells (3).

In schizogony, within a single shared cytoplasm, a cell must replicate one genome at first and then as many as dozen genomes just a few hours later, with varying degrees of asynchrony. How this is organised is now being studied in detail at the cell-biological level (4–6) but at the molecular level much remains unknown. Most fundamentally, we were interested to know how replication origins are specified and controlled in a genome undergoing several rounds of asynchronous replication and repeated karyokinesis. Our earlier work, using DNA fibre labelling, showed that replication origin spacing changes across the course of schizogony, with closer-spaced origins occurring in later rounds of replication (7). Some malaria parasites including *P. falciparum* also have very unusual genome compositions at over 80% A/T (8), the most biased genomes ever sequenced, while other *Plasmodium* species, with similar lifecycles and cell biology, have genomes of only ∼60% A/T (9). We recently made a preliminary study, again via DNA fibre labelling, of whether origins are specified and controlled similarly in genomes whose nucleotide composition varies so markedly. Origin spacing was identical during schizogony in A/T-biased versus A/T-balanced species (6). However, a key shortcoming of DNA fibre labelling is that it does not identify the underlying DNA sequence, so we could not establish whether replication origins are specified by a sequence motif, whether this might vary in different *Plasmodium* genomes, and what might determine the increased density of replication origin firing in late schizogony.

Replication origin specification has been thoroughly studied in model eukaryotes such as *Saccharomyces cerevisiae* (10), where a complex of proteins based on the origin-of-replication (ORC) complex binds to DNA at specific sites called autonomous replication sequences (ARS). Once these origins are ‘licenced’, they can initiate DNA replication during S-phase (11). In other model eukaryotes including *Schizosaccharomyces pombe*, *Drosophila* and *Xenopus*, the replication machinery is broadly conserved but ARSs are not. Research continues into factors that may specify an origin in these species, from DNA sequence to DNA secondary structure to chromatin architecture (12). In *S. pombe*, for example, A/T-rich sequences are favoured (13), while in human cells, G-rich G-quadruplex-forming sequences are implicated (14).

In *Plasmodium*, the replication machinery is again broadly conserved, albeit with some protein components missing or unidentified (reviewed in Matthews *et al.*, 2018 (15)). There is limited functional evidence for an ARS, although one computational study has suggested that it may exist (16). ORC subunit 1 (ORC1) plays an important role in DNA replication and has been identified in *P. falciparum* alongside several other ORC subunits (17). In mammals, ORC1 is strongly bound to DNA prior to replication and eventually loses its affinity, or in some cases is degraded, once replication ensues (reviewed in DePamphilis *et al.* (18)) making this subunit ideal for genome-wide mapping of potential replication origins (19).

In this study, we mapped origins of replication empirically in *P. falciparum* for the first time. In model systems this has been achieved in various ways, including chromatin immunoprecipitation (ChIP) for proteins such as ORC1, and mapping of nascent DNA synthesis by diverse means, including purifying modified nucleotides incorporated into nascent DNA (20). However, such mapping has never been achieved, to our knowledge, in an apicomplexan parasite that does not replicate via binary fission. The constraints of schizogony could be fundamentally different, requiring multiple simultaneous rounds of genome replication, under high levels of oxidative stress and in the absence of certain DNA repair pathways and checkpoints.

We have used ORC1-ChIP to map all potential origin sites and combined this with a novel application of a recently published nanopore method (21–25) to map *activated* origins and compare them with potential origins. This novel method exploits long-read sequencing to simultaneously detect and map modified nucleotides in nascent DNA strands. We have developed this into a powerful two-nucleotide protocol with new DNAscent software that distinguishes sequential pulses of thymidine analogues in single molecules. This provides high resolution and critical insight into replication origin usage in this unusual eukaryote: the most A/T-rich genome in which DNA replication has ever been mapped, and also the first apicomplexan that uses schizogony as a mode of cell division. It also lays vital groundwork for future studies comparing origin usage in different *Plasmodium* species and in different stages of the lifecycle.

## MATERIALS AND METHODS

### Parasites

Continuous *P. falciparum* 3D7 cultures were grown in custom made RPMI 1640 (with 2.3 g/l sodium bicarbonate, 4 g/l dextrose, 5.957 g/l HEPES, 0.05 g/l hypoxanthine, 5 g/l Albumax II, Invitrogen) and 5% pooled human serum (NHS Blood and Transplant), i.e. complete media, in 4% haematocrit O + human red blood cells (NHS Blood and Transplant) in 3% oxygen, 5% CO_2_ and 92% nitrogen gas mixture at 37°C. Parasites were initially synchronised with 5% sorbitol (w/v in water) and were allowed to grow to late schizont stage. Schizonts were harvested using a 65% Percoll (GE Healthcare) gradient (v/v in PBS) and incubated for 2 h in complete media with 1.5 μM Compound 2 (LifeArc) (26). Compound 2 was washed off with RPMI and mature schizonts were allowed to reinvade in 25% haematocrit red blood cells in 5 ml of complete media in the abovementioned gas mixture at 37°C for 1 h at 220 rpm. The remaining schizonts were removed using 5% sorbitol to produce a tightly synchronised culture (0–1 h post invasion, referred to as 0 hpi).

### Generation of genetically modified parasites

*P. falciparum* 3D7 *orc1* (PF3D7_1203000) was C-terminally tagged with 3xHA using the selection linked integration (SLI) technique (27). Approximately 500 bp from the 3’ end of the *orc1* gene (excluding the stop codon) was amplified and cloned between the NotI and KpnI sites of a pSLI 3’ HA-Neomycin resistance tagging vector with a human DHFR selection marker (Supplementary Figure S1a). The plasmid (100 μg) was electroporated into a predominantly ring-stage culture and was selected initially with 5 μM WR99210 (Jacobus Pharmaceutical) followed by 400 μg/ml of G418 (Merck). Successful transfection and gene tagging was confirmed by PCR and western blot (Supplementary Figure S2a and b, Supplementary Table S1). A stable line with mostly ring-stage parasites was transfected with 100 μg of blasticidin-selectable plasmid with a thymidine kinase expression cassette (pTK-BSD) to allow episomal thymidine kinase expression and incorporation of nucleotide analogues during DNA replication (Supplementary Figure S1b). Successful transfection was selected with 2.5 μg/ml of blasticidin (Gibco) and was confirmed by ELISA and immunofluorescence for the presence of incorporated BrdU in parasite DNA (28). The resulting modified parasite line, i.e. *P. falciparum* 3D7 ORC1-3xHA + pTK-BSD, was utilised for subsequent ChIP and immunofluorescence experiments. Crude parasite protein lysates from tightly synchronised parasites were fractionated into cytosolic, nuclear soluble and nuclear insoluble fractions as previously described in Voss *et al.* (29). Briefly, saponin-lysed parasites were incubated for 5 min in ice-cold lysis buffer containing 20 mM HEPES, pH 7.8, 10 mM KCl, 1 mM EDTA, 1 mM DTT, 1 mM PMSF, 0.65% Nonidet P-40. Cytoplasmic protein fraction (supernatant) was obtained by centrifugation at 2500g for 5 min. The pellet was then incubated in extraction buffer containing 20 mM HEPES, pH 7.8, 800 mM KCl, 1 mM EDTA, 1 mM DTT, 1 mM PMSF, 1× Pierce protease inhibitor (ThermoFisher) shaking at 2000 rpm at 4°C for 30 min. Centrifugation was done at 13 000g for 30 min to obtain the soluble and insoluble nuclear proteins (supernatant and pellet, respectively). Western blot of the fractionated samples was probed using rat anti-HA (Roche) antibodies diluted 1:1000 in block (1% BSA with 0.1% Tween 20). As control, the western blot membrane was also probed using mouse monoclonal anti-*P. falciparum* GAPDH antibody obtained from The European Malaria Reagent Repository (clone 13.3), and rabbit polyclonal anti-histone H4 antibody (abcam, ab10158).

### Immunofluorescence

Tightly synchronised *P. falciparum* 3D7 ORC1-3xHA + pTK-BSD culture was incubated in 100 μM BrdU (Sigma) 30 min prior to collection of samples for immunofluorescence staining. Parasite smears were done every 4 hours from 18 hpi to 46 hpi. Thick smears were fixed with 4% formaldehyde in PBS for 10 min followed by permeabilization in 0.2% Triton-X100 for 15 min. Slides were blocked in 1% BSA with 0.1% Tween-20 for 1 hour. Primary antibody labelling was done for 1 hour using mouse anti-BrdU (GE Healthcare, clone BU-1) and rat anti-HA (Roche, clone 3F10) antibodies diluted 1:500 in 1 U/ml DNAse I in Tris Buffered Saline containing 1% BSA (GE Healthcare). Three 5-min washes with block (1% BSA with 0.1% Tween-20) were done prior to incubation in secondary antibodies (ThermoFisher Alexafluor goat anti-mouse 488 and Alexafluor goat anti-rat 594) in block at 1:1000 dilution for 1 h. Three 5-min final washes were done with block, with the second wash replaced with DAPI (ThermoFisher) at 2 μg/ml in PBS. All incubation steps were done at room temperature. Slides were cured using ProLong Diamond Antifade Mountant (Invitrogen) and were stored at 4°C prior to visualisation.

Images were acquired using a Nikon Microphot SA fluorescence light microscope with a Qimaging Retiga R6 camera at 1000× magnification. Images were taken from highest to lowest emission wavelength to avoid bleed through. All images (including images taken from different channels) were taken using the following parameters: 2 s exposure time, no binning, readout of 50 MHz, 14 bits, system gain 1, and mono colour output. Saturation histogram was adjusted to ensure that none of the fluorescence signal was missed. ImageJ (30) was used to convert images to 32-bit prior to the identification and creation of regions of interest (ROIs) on nuclear signal in the DAPI channel images. These ROIs where then used to measure the area and integrated signal density, i.e. as the sum of the grey values of all pixels within a given area (30), in all images taken using the different fluorescent channels. Background subtraction was not done as all images were obtained using the same parameters. The resulting data were analysed and plotted using GraphPad Prism v9.3.1. Representative images were pseudo-coloured and merged using ImageJ.

### Chromatin immunoprecipitation

Cultures of 2.0 × 10^9^ and 2.4 × 10^9^ tightly synchronized parasites at 24 and 30 hpi, respectively, were used for ChIP. Chromatin was crosslinked with 1% formaldehyde in culture media for 10 min at 37°C, then quenched with glycine at a final concentration of 0.125 M on ice. Samples from here on were maintained on ice. Parasites were extracted by lysis with 0.05% saponin in PBS. Nuclei were extracted by gentle homogenisation in cell lysis buffer (10 mM Tris pH 8.0, 3 mM MgCl_2_, 0.2% NP-40, 1× Pierce protease inhibitor (ThermoFisher)) and centrifugation at 2000 rpm for 10 min in 0.25 M sucrose cushion in cell lysis buffer. Harvested nuclei were snap-frozen in 20% glycerol in cell lysis buffer. Samples were resuspended in shearing buffer (Tris–HCl 10 mM, EDTA 1 mM, 0.1% SDS, pH 7.6) to a final volume of 1 ml and sonicated in a milliTUBE-1 with AFA fibre (Covaris) using a Covaris M220 sonicator with the following settings:

Duty factor: 5%

Temperature: min: 5.0°C; setpoint: 7.0°C; max: 9.0°C

Peak power: 75.0 W

Cycle/burst: 200

Processing time: 2700 s (45 min)

Each ChIP reaction was set up with 500 ng sonicated chromatin in incubation buffer (0.15% SDS, 1% Triton-X100, 150 mM NaCl, 1 mM EDTA, 0.5 mM EGTA, 1× protease inhibitor (Sigma-Aldrich), 20 mM HEPES, pH 7.4) with 400 ng of anti-HA (Roche, clone 3F10), together with 10 μl protA and 10 μl protG Dynabeads suspension (ThermoFisher). For each sample, eight ChIP reactions were prepared and incubated overnight rotating at 4°C. Beads were washed twice with wash buffer 1 (0.1% SDS, 0.1% DOC, 1% Triton-X100, 150 mM NaCl, 1 mM EDTA, 0.5 mM EGTA, 20 mM HEPES, pH 7.4), once with wash buffer 2 (0.1% SDS, 0.1% DOC, 1% Triton-X100, 500 mM NaCl, 1 mM EDTA, 0.5 mM EGTA, 20 mM HEPES, pH 7.4), once with wash buffer 3 (250 mM LiCl, 0.5% DOC, 0.5% NP-40, 1 mM EDTA, 0.5 mM EGTA, 20 mM HEPES, pH 7.4) and twice with wash buffer 4 (1 mM EDTA, 0.5 mM EGTA, 20 mM HEPES, pH 7.4). Each wash step was performed for 5 min rotating at 4°C. Immunoprecipitated chromatin was eluted in elution buffer (1% SDS, 0.1M NaHCO_3_) at room temperature for 20 min. The eluted chromatin samples and the corresponding input samples (sonicated input chromatin containing 500 ng DNA) were de-crosslinked in 1% SDS/0.1M NaHCO_3_/1M NaCl at 45°C for overnight while shaking, followed by column purification (PCR Purification Kit, Qiagen) and elution in 20 μl EB buffer. The obtained ChIP-ed DNA fragments were used to generate Illumina sequencing libraries according to Filarsky *et al.* (31). In brief, 5 ng of α-HA ChIP or input DNA were end-repaired with T4 DNA polymerase (NEB), Klenow DNA polymerase (NEB), and T4 Polynucleotide Kinase (NEB). The 3’ends of end-repaired DNA were extended with an A- overhang with 3’ to 5’ exonuclease-deficient Klenow DNA polymerase (NEB). The resulting fragments were ligated to Nextflex adapters (Bio Scientific) with the use of T4 DNA ligase (Promega). The libraries were amplified using an AT-rich optimized KAPA protocol using KAPA HiFi HotStart ready mix (KAPA Biosystems), NextFlex primer mix (Bio Scientific) with the following PCR program: 98°C for 2 min; four cycles of 98°C for 20 s, 62°C for 3 min; 62°C for 5 min. The fragments originating from mono-nucleosomes + 125 bp NextFlex adapter were selected using 2% E-Gel Size Select agarose gels (Invitrogen) and amplified by PCR for 8 cycles using the above conditions. Libraries were purified and adapter dimers removed with Agencourt AMPure XP beads purification using a 1:1 library:beads ratio (Beckman Coulter). ChIP-seq libraries were sequenced on the Illumina NextSeq 500 system with a 20% phiX spike-in (Illumina) to generate 75 bp single-end reads (NextSeq 500/550 High Output v2 kit).

Sequencing reads were mapped against the PlasmoDB v26 of the *P. falciparum* genome (version 3) (32) utilising the Burrows-Wheeler Alignment tool (v0.7.17) (33). Mapped reads were filtered to mapping quality ≥30 (SAMtools v1.7) (34) and only uniquely mapped reads were used for further analysis.

### Datasets for EdU and BrdU model training

To produce training data sets for the DNAscent algorithm, tightly synchronised thymidine kinase-expressing *P. falciparum* 3D7 culture (28) was incubated with either 20 μM EdU (ThermoFisher) or 200 μM BrdU (Sigma) for 1 h. Parasite DNA was then harvested using a DNeasy Blood and Tissue DNA extraction kit (Qiagen). DNA barcoding was done on 1 μg of high molecular weight genomic DNA using Oxford Nanopore ligation sequencing kit (SQK-LSK109) and barcode expansion kit (EXP-NBD104). Sequencing was done using a Nanopore MinION device with R9.4.1 flowcells for 72 h or until the pores in the flowcell have been fully depleted. High molecular weight parasite genomic DNA was extracted using a Qiagen MagAttract kit and stored at 4°C. Long DNA fragments were enriched using SRE/SRE-XL short read eliminator buffer (Circulomics) prior to library preparation and nanopore sequencing.

### Datasets for single-molecule origin and fork calling

To produce data sets for the identification of active replication forks and origins, tightly synchronised parasites were incubated in 20 μM EdU for 7.5 min, then 200 μM BrdU for another 7.5 min, followed by 2 mM thymidine for 1 hour and 45 min prior to parasite harvest. Nascent DNA labelling was done at 30 and 36 hpi. High molecular weight genomic DNA was extracted and processed as mentioned in the previous paragraph.

### DNAscent software development and model training

Given the stochasticity of DNA replication in erythrocytic schizogony, we expected only a minority of sequenced reads in the training set to be analogue-substituted. While DNAscent v2.0.2 (22) was trained to identify BrdU, we found that the current shifts caused by BrdU and EdU were sufficiently similar that DNAscent v2.0.2 often called EdU as BrdU in EdU-substituted DNA (Supplementary Figure S3). DNAscent v2.0.2 was therefore used to identify and pull out analogue-substituted reads that were suitable for training. Reads where DNAscent v2.0.2 called BrdU with probability greater than 0.5 for at least 20% of thymidine positions were considered to be analogue-substituted, resulting in 6917 BrdU-substituted reads and 3162 EdU-substituted reads. Training reads were split into 4 kb segments and augmented with thymidine reads as in Boemo, 2021 (22). The neural network architecture used was identical to that of Boemo, 2021 (22), with the exception that the dimension of the time-distributed softmax layer in was increased from two to three to reflect the three classification categories (thymidine, BrdU, or EdU). The model was trained for 21 epochs on 15 000 EdU-augmented segments, 15 000 BrdU-augmented segments and 10 000 thymidine-only segments. ROC curves were computed in *S. cerevisiae* reads from Muller *et al.* (21) to show that the new model, despite calling two thymidine analogues instead of one, had similar BrdU-calling accuracy and lower false positive rate compared to the BrdU-only model used in DNAscent v2.0.2 (Supplementary Figure S4). For the new model, ROC curves computed on *P. falciparum* test reads showed BrdU- and EdU-calling performance were similar to each other, the frequency with which the model would confuse BrdU for EdU (and vice versa) was low, and that the model had a very low false positive rate of approximate 1-in-700 thymidines (Supplementary Figure S5).To make replication origin, fork, and termination calls from patterns of analogue calls, the rate analogue incorporation in analogue-substituted regions was measured by K-means clustering (*K* = 2) the rate of analogue substitution in consecutive 2 kb segments. The rate of analogue incorporation in analogue-substituted regions was taken to be the greater of the two centroid means. Reads were then segmented with a DBSCAN algorithm (epsilon = 1000 bp; minimum density = analogue centroid mean – 1 standard deviation). Different analogue segments closer than 5 kb apart were matched into forks and origins were called as the genomic regions between matched left- and rightward-moving forks.

### Data analysis

ChIP and DNAscent origin log_2_ ratios were calculated using deepTools 3.5.0 bamCompare by normalising reads against input/background reads, with a pseudocount of +1 and a bin size of 50 bp (35). Normalisation was done separately for the two ChIP replicates. Nanopore read normalisation was done on a per barcode basis and log2ratios were summed by timepoint. MACS2 v2.1.1 was used for ChIP peak calling with *P*-value cut-off of 1e–3 (36). BEDtools v2.30.0 (37) was used for file format conversions, comparison of multiple data sets, and identifying protein-coding sequences overlapping with areas of interest in the genome (bedtools intersect). GC-bias (computeGCBias), Spearman R correlation analyses between multiple bigwig files (multiBigwigSummary, bin size of 100 bp), and matrix plots (computeMatrix) were done using deepTools 3.5.0. We have classified correlation coefficient values as 0–0.19 as very weak, 0.20–0.39 as weak, 0.40–0.59 as moderate, 0.60–0.79 as strong and 0.80–1 as very strong correlation based on recommended the classification by Evans (1996) (38). Fork speed calculation were done on all forks identified by DNAscent that are not located at the Nanopore read ends, or joint at the replication origin or termination site. Genome-wide mean fork speed *z*-score per 100 bp bin was calculated as a metric to determine the deviation of the mean fork speed at any given region in the genome from the overall mean fork speed. Fork speed was calculated by dividing the fork length (in kb) by 15 min (the duration of the pulse) giving the fork speed in kb/min. Fork speed *z*-score was then calculated using the formula:


$$\begin{equation*}\frac{{{\rm fork}\,{\rm speed }- {\rm overall}\,{\rm mean}\,{\rm fork}\,{\rm speed}}}{{{\rm overall}\,{\rm standard}\,{\rm deviation}}}\end{equation*}$$

Fork speed *z*-score per 100 bp was determined using bedtools closest to identify forks that overlap any given 100 bp window in the genome, and bedtools merge to calculate the mean per window.

*De novo* motif search was done on ORC1 ChIP summits ±50 bp using HOMER motif analysis software v4.11.1 (39). Search parameters included motif lengths of 8, 10, 12, 15, 20, 25 and 30 bp and sequence content G/C normalisation. The calculation of hypergeometric p-values was done in reference to motif occurrence in preparsed background sequences. Similar motif search parameters were used on DNAscent origin centres ±50 bp. Motifs with *P*-values ≤1e–12 were considered significant. Motif weighted G/C content was calculated as the sum of the individual probability of a G or C in the motif sequence divided by the sum of the probabilities for all nucleotides. G4Hunter (40) was used to identify G-quadruplex motifs in ORC1 ChIP peaks with the following parameters: window size of 25 and threshold of 1.7. Enrichment in ChIP peak sequences was calculated as the total number of identified G4 per bp divided by the total number of G4 (+1, to avoid a zero denominator) per bp in the entire genome.

Data on predicted AP2 transcription binding sites (41) and genome-wide HP1 log_2_ratio (42) were downloaded from PlasmoDB (17). Other data sets utilised in the analyses were RNA-seq/ATAC-seq (43) and histone placements (44). Gene ontology (GO) terms were obtained from PlasmoDB (17) and were collated and summarised using Python. Bigwig, bedgraph and bed files were visualised using Integrative Genomics Viewer (IGV) version 2.11.2 (45) or UCSC Genome browser (46). D’Agostino and Pearson's test for normality, Mann–Whitney *U* test for significant difference, and Spearman *R* test for correlation between smaller data sets were done using Scipy 0.10.1 (47).

To call replication forks and origins on single molecules, Oxford Nanopore sequencing reads were basecalled and demultiplexed with Guppy (v5.0.11) and aligned to the *P. falciparum* 3D7 ASM276v2 assembly (https://www.ncbi.nlm.nih.gov/assembly/GCF_000002765.4/, version 3 of the genome sequence) with minimap2 (v2.17-r941). Only sequences with an alignment length ≥10 kb and mapping quality ≥20 were analysed. The probability of BrdU and EdU at thymidine positions along each read was assigned by DNAscent detect, and these probabilities were parsed into replication fork and origin calls by DNAscent forkSense. Bedgraphs of base analogue calls on single molecules were generated using the dnascent2bedgraph utility.

## RESULTS

### Distribution of ORC1 is independent of active DNA replication during S-phase in *P. Falciparum*

To examine the localisation and intensity of the origin recognition complex subunit 1 (ORC1) in relation to DNA replication in *P. falciparum*, we first C-terminally tagged the *orc1* gene with 3xHA, then transfected a thymidine kinase-expressing plasmid. Thymidine kinase allows the parasites to salvage thymidine analogues such as bromo-deoxyuridine (BrdU) or ethynyl-deoxyuridine (EdU) without affecting parasite cell-cycle and survival in the short-term (28). Every 4 h, from 18 hpi to 46 hpi, an aliquot of a tightly synchronised culture was labelled with BrdU for 30 min, then fixed and processed for microscopy. DAPI was used to detect the parasite nuclei, while anti-BrdU and anti-HA were used to detect newly replicated DNA and ORC1, respectively.

**Figure 1.**
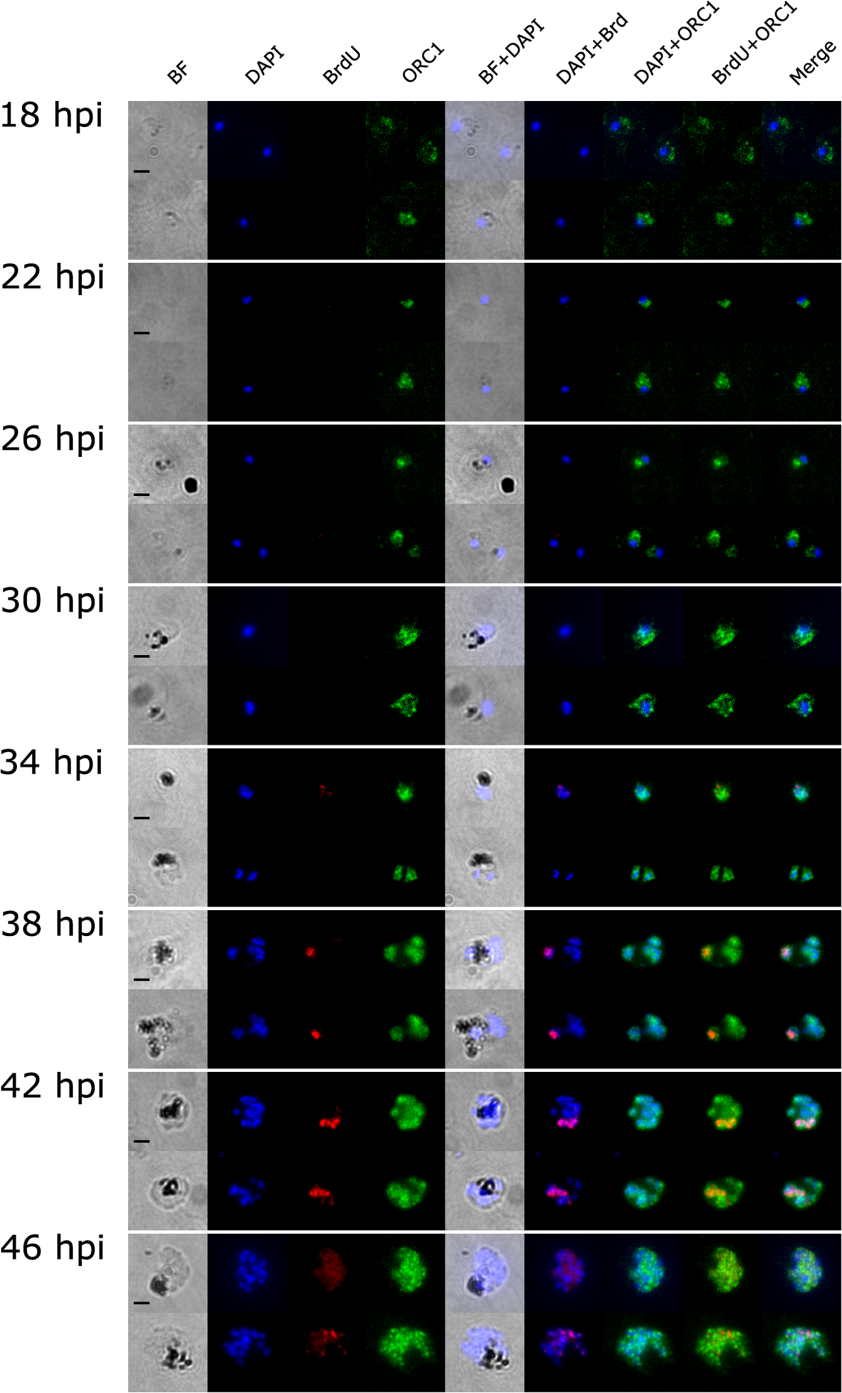
Representative immunofluorescence images showing ORC1 distribution to be independent of BrdU labelling in *P. falciparum* nuclei. Synchronised *P. falciparum* 3D7 ORC1-3xHA + pTK-BSD was incubated in BrdU for 30 mins at 4-hour intervals from 18 to 46 hpi. ORC1 (in green) and BrdU (in red) were probed using anti-HA and anti-BrdU antibodies, respectively. DNA was stained with DAPI (blue). Scale bar (2 μm) applies to all images.

**Figure 2.**
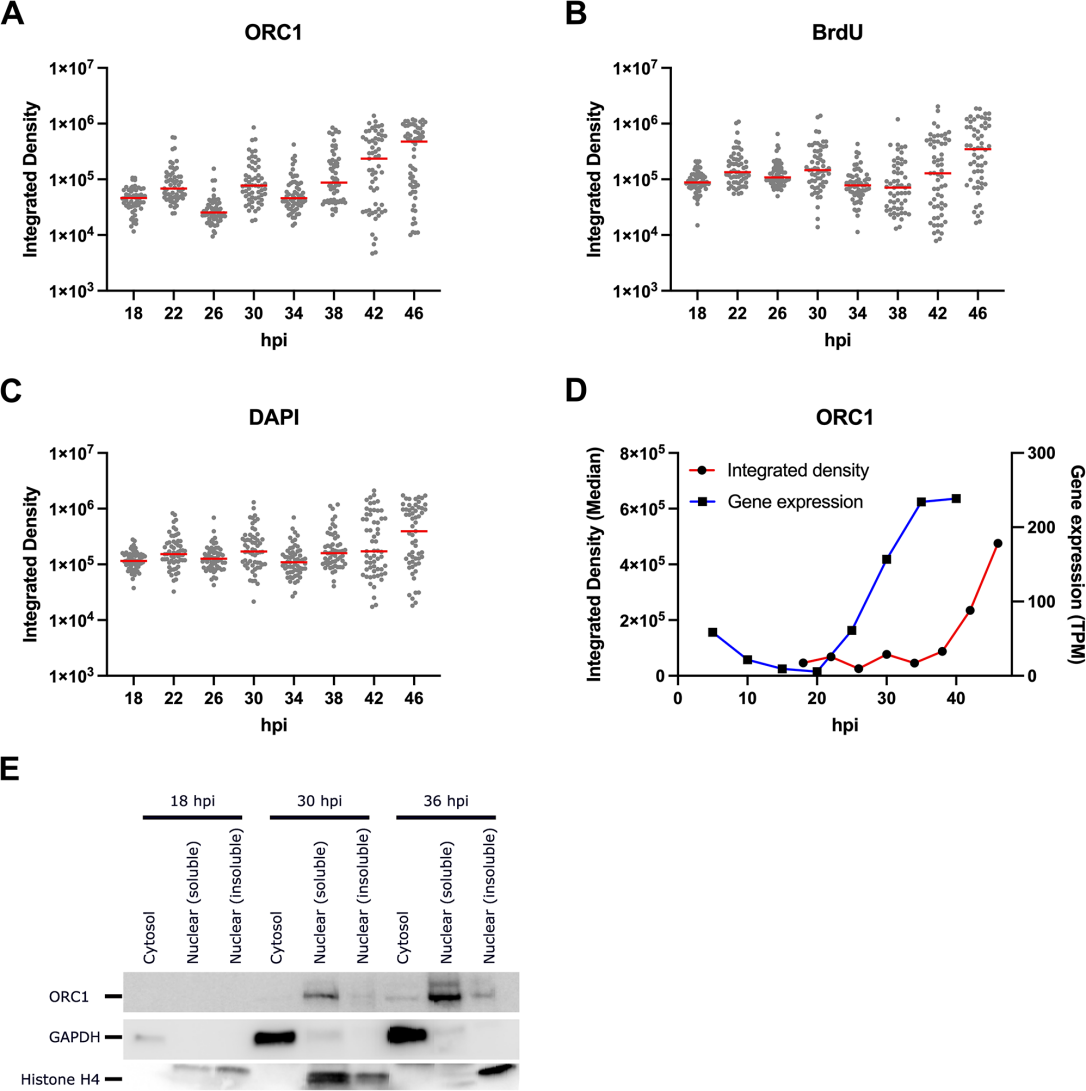
ORC1 abundance increases throughout S phase and follows changes in its transcript abundance. Integrated densities of (**A**) ORC1, (**B**) BrdU and (**C**) DAPI in tightly synchronised *P. falciparum* 3D7 ORC1-3xHA + pTK-BSD from 18 to 46 h post invasion (hpi), with the y-axes shown in log scale to show differences in median integrated densities between time points. The median values are represented by red bars in the dot plots (*n* = 56–62 cells per timepoint). (**D**) Median ORC1 integrated density in comparison with *orc1* gene expression in transcripts per million (TPM) (43). (**E**) Western blot from fractionated crude protein lysates from synchronised *P. falciparum* 3D7 ORC1-3xHA + pTK-BSD parasites showing ORC1, GAPDH and Histone H4 at 18, 30 and 36 hpi.

Prior to DNA replication and the formation of the first daughter nucleus, ORC1 was distributed throughout the whole cell, and not strictly localised in the parasite nucleus (Figure 1, 18 hpi). As the ring stage progressed (22 and 26 hpi), it was found generally in the nuclear periphery, consistent with Mancio-Silva *et al.* (48). As soon as replication occurred (30 hpi), ORC1 was in closer proximity with the nucleus, and eventually formed distinct spots on the nucleus and its periphery towards the latter part of schizogony (42 hpi onwards). ORC1 signal was not present in greater amounts in actively replicating nuclei, marked by BrdU (Figure 1, see cell at 34 hpi in which neither of the 2 nuclei has replicated in the prior 30 min but ORC1 remains clearly nuclear, and cells at 38 hpi in which only a minority of nuclei are replicating but ORC1 is equally present in all nuclei). Quantitative analysis of the signal intensity of BrdU and ORC1 (*n* = 56–62 cells per timepoint), showed slight fluctuations in ORC1 signal from 18 to 34 hpi, followed by an increase reaching its peak at 46 hpi (Figure 2A, D). These changes in ORC1 intensity coincided with changes in DNA replication as shown by increases in BrdU and DAPI signal (Figure 2B, C). The temporal pattern of ORC1 intensity followed *orc1* expression pattern at the RNA level, with a delay of several hours. The lowest transcription, observed at 20 hpi, and the peak between 35 and 40 hpi (43), were followed by ORC1 intensity at the protein level (Figure 2D, E).

### ORC1 chromatin immunoprecipitation reveals that potential origins are densely distributed throughout the genome

**Figure 3.**
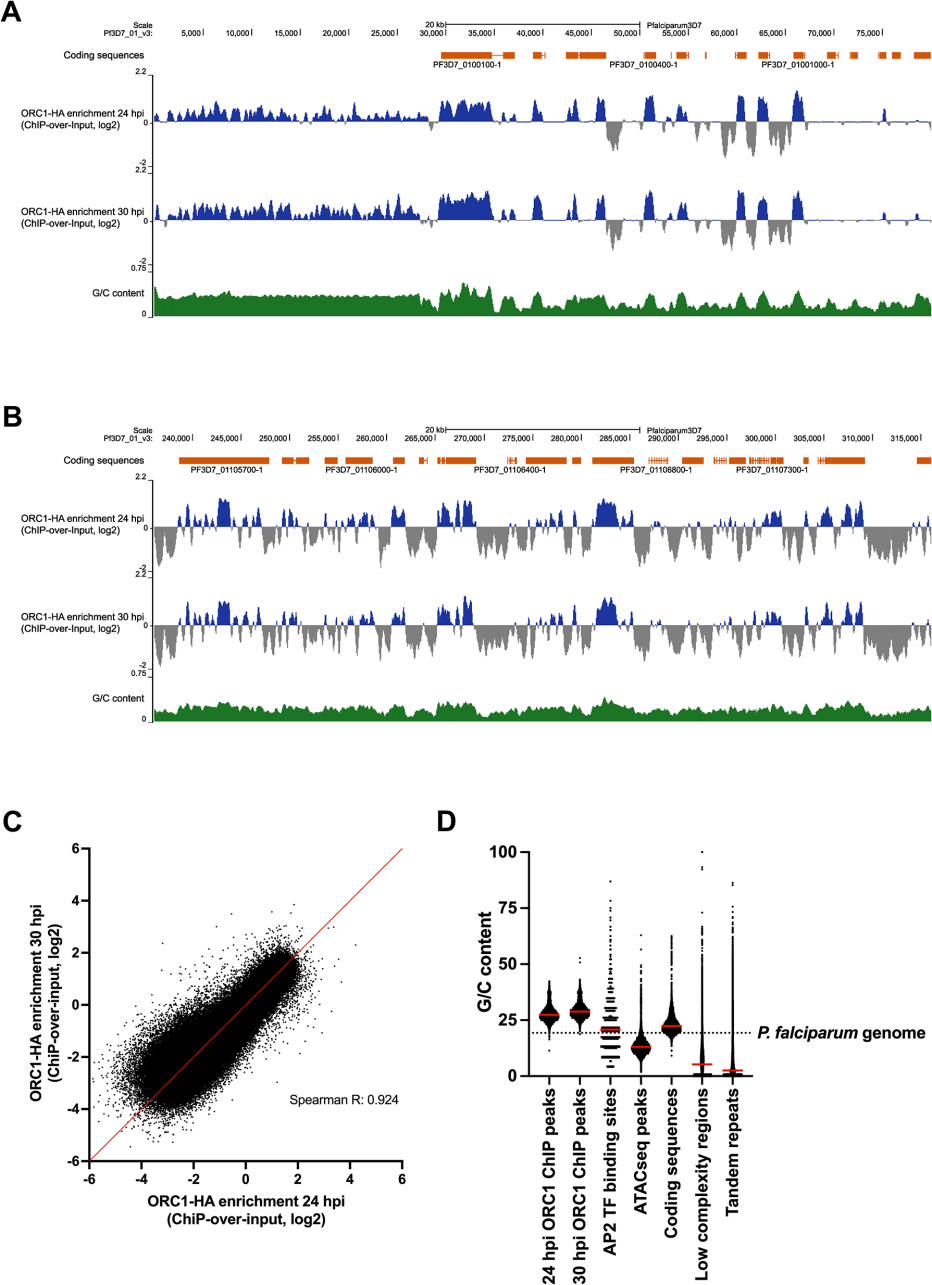
ORC1 ChIP-seq shows consistent ORC1 localisation in relatively high G/C regions. (**A**) ORC1 ChIP log_2_ratios (normalised against input) at 24 and 30 hpi (middle two panels, in blue) on the first 80 kb of chromosome 1 are visualised here alongside coding sequences (top panel, in orange) and G/C content (bottom panel, in green). (**B**) ORC1 ChIP log2ratios at 24 and 30 hpi in a non-telomeric region. (**C**) Scatterplot where points are the average log2ratios of ORC1 ChIP data over each non-overlapping 100 bp window in the genome at 24 hpi (x-axis) and 30 hpi (y-axis). The line where *x* = *y* is show in red. (**D**) Comparison between the G/C content at ORC1 ChIP-seq peaks and the G/C content at ATAC-seq peaks, predicted binding sites of AP2 transcription factors, coding sequences, low complexity regions, and tandem repeats. The overall G/C content of *P. falciparum* is represented by the dashed line while red bars represent the median G/C content.

ORC1 plays an important role in DNA replication and has been identified in *P. falciparum* alongside several other ORC subunits (17,49). ORC1 binds to replication initiation sites on the genome in preparation for S-phase (18). To identify potential replication initiation sites, we analysed the genomic placement of ORC1 through chromatin immunoprecipitation-sequencing (ChIP-seq) at two different pre-S-phase timepoints (24 and 30 hpi, Figure 3A, B). Data from the two timepoints showed near identical results (Spearman *R*: 0.924) (Figure 3C). There was a strikingly high density of ORC1 summits, ∼1/kb (median inter-summit distance of 862.5 bp, Supplementary Table S2) and peaks showed a preference for relatively high G/C sequences (Figure 3A and B, median G/C content of 27.0% and 28.4% at 24 and 30 hpi, respectively). ORC1 was enriched in, but not limited to, the coding sequences which are likewise higher in G/C than the total genome (Figure 3d). Furthermore, we observed ORC1 enrichment in sub-telomeric regions, consistent with previously published data (48,50) (Figure 3A). To ensure that the results were ORC1-specific, peak-calling analysis was done using the same parameters on a control data set, i.e. anti-HA ChIP on a non-tagged parasite line. In comparison with the 24 and 30 hpi data sets, where a total of 8843 and 6785 peaks were called respectively, only 33 peaks were called on the control data set, validating the specificity of the ORC1 ChIP peaks (Supplementary Tables S3–S5).

ORC deposition is not sequence-specific in most organisms, but the *S. cerevisiae* autonomously replicating sequence (ARS) is an exception, and similar sequences have been proposed to act as ORC landing pads in *P. falciparum* (16). We therefore conducted a *de novo* motif search on ORC1 ChIP sequences ±50 bp from the summits using HOMER motif analysis software. We identified 12 unique overrepresented motifs where ORC1 was enriched at 24 and 30 hpi (Supplementary Table S6); however these motifs were only moderately enriched and are unlikely to serve a similar function as ARS. In contrast to the G/C-poor ARS, G-rich sequences that can form G-quadruplexes (G4s) have been proposed to promote ORC binding in human cells (51,52). Therefore, we also calculated the enrichment of G4-forming motifs in ORC1 ChIP peaks using G4 Hunter (40,53). Compared with the overall occurrence of G4-forming motifs in the whole *P. falciparum* 3D7 genome, there was a small enrichment of 2.05- and 2.82-fold in ORC1 ChIP peak sequences at 24 and 30 hpi, respectively (Supplementary Table S7).

Finally, the chromatin landscape has been proposed to influence ORC distribution in some systems (20), so we compared ORC1’s whole genome distribution with previously published epigenetic landscapes such as variant and modified histone placements, chromatin accessibility and predicted binding sites of the apicomplexan apetala2 (ApiAP2) transcription factors (41). We found no correlation or a very weak negative correlation with most histone and modified-histone distributions (Spearman *R*: –0.153 to 0.033). Interestingly, there was more negative correlation between ORC1 placement and predicted AP2 binding sites (Spearman *R*: –0.208 to –0.192) and a moderate-to-strong negative correlation with accessible chromatin (Spearman *R*: –0.630 to –0.283) (Supplementary Table S8). Open chromatin identified through the Assay for Transposase-Accessible Chromatin (ATAC-seq) correlates with transcription factor binding (43). This suggests a potential inverse relationship between replication origin placement and transcription dynamics. However, since ORC1 was generally associated with areas of high-G/C sequence and with coding sequences, the G/C content at the associated genomic locations could be a potential confounding factor (median G/C at ATAC-seq peaks: 13.1%).

We also compared ORC1 distribution with heterochromatin protein 1 (HP1) since these proteins co-localise or interact with each other in *Plasmodium* and other organisms (50,54–56). Consistently, we observed weak to moderate positive correlation between ORC1 and HP1 (Spearman *R*: 0.3402–0.5737). ORC1 and HP1 are both associated with telomeres (48,57), but we also calculated correlation between ORC1 and HP1 outside the telomeric regions and still found positive correlation (Spearman *R*: 0.2555–0.5095) (Supplementary Table S8).

### Detection of replication origin activity with single-molecule resolution

**Figure 4.**
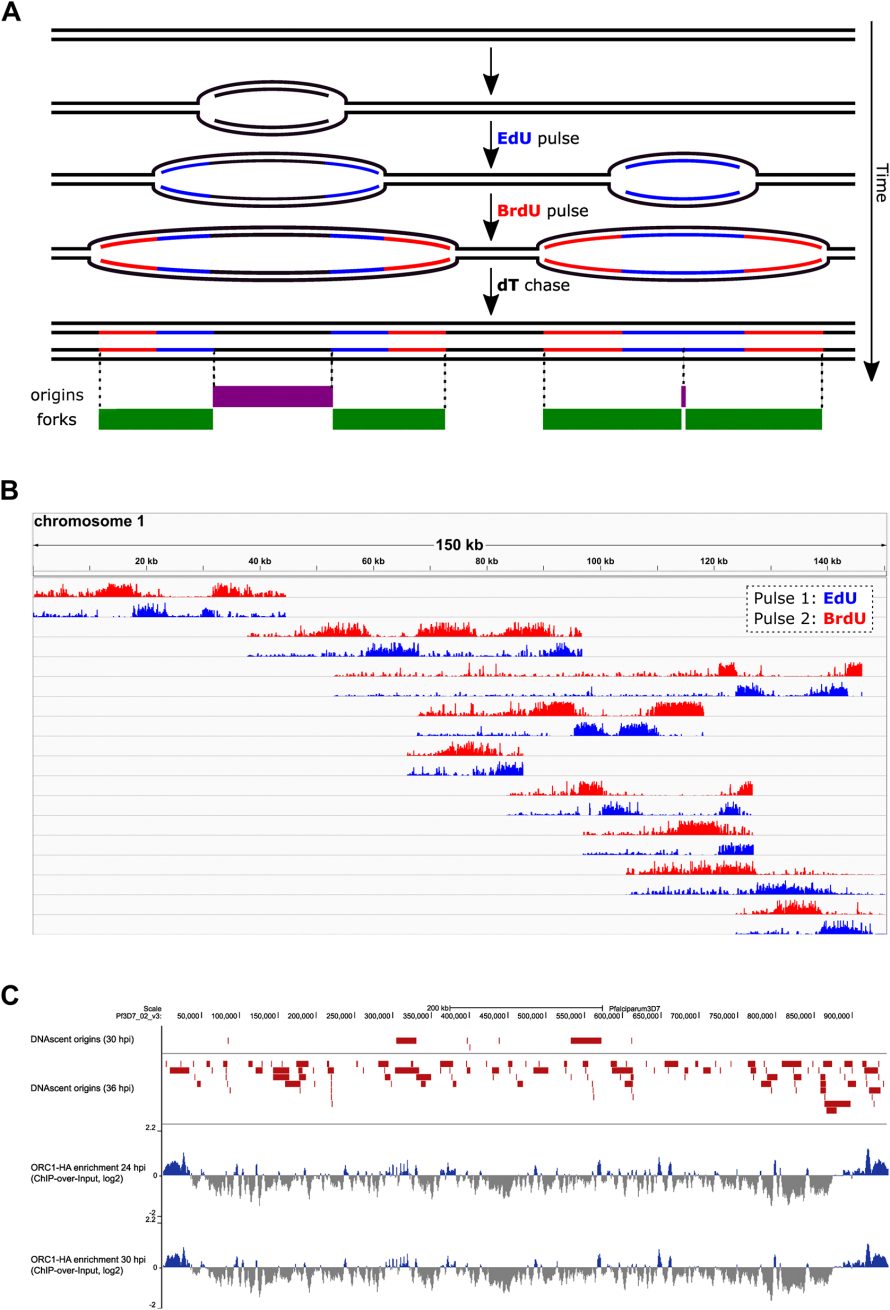
DNAscent v3.0.2 identifies actively replicating DNA and replication origins. (**A**) Schematic of the pulse-labelling protocol used to prepare sequencing reads for analysis by DNAscent v3.0.2. The thymidine analogue EdU (blue) is pulsed into replicating DNA where it is incorporated into the nascent strand by replication forks. This is followed by the pulse of a second thymidine analogue BrdU (red) to indicate fork direction (as in established DNA fibre-labelling protocols), which is in turn followed by a thymidine chase. Coordinates of origins and forks called by the DNAscent software are represented in purple and green, respectively. (**B**) Nine single molecules sequenced through nanopore that mapped to *P. falciparum* chromosome 1 and were analysed with DNAscent v3.02. Each molecule is represented as two bedgraph tracks showing the probability of BrdU (red) and EdU (blue) called by DNAscent v3.0.2 at each thymidine position in the read. (**C**) Visualisation showing fired origins called by DNAscent at 30 and 36 hpi on chromosome 2 (red) with ORC1 ChIP peaks (positive peaks in blue, negative peaks in grey) for comparison.

Our ORC1 ChIP-seq data identified a high density of ORC1 placement throughout the genome (∼1 per kb). This is a population-level experiment, so every site may not be occupied in every parasite, but nevertheless it was notable that our previous data showed a lower density of active origins on actively replicating DNA fibres (∼1 per 65 kb) (7). This difference in the density of ORC1 placement and active origin firing suggests that parasites license a large excess of origins and fire only a small subset, similar to other organisms (58,59). To verify this, we used long-read Oxford Nanopore sequencing of genomic DNA to identify active replication with sequence-specificity and single-molecule resolution. *P. falciparum* parasites at early and mid S-phase were sequentially pulse-labelled with EdU and BrdU, for a total of 15 min followed by high-molecular-weight DNA preparation and sequencing. The analogues would thus mark active DNA replication forks, allowing us to confidently identify replication fork directionality and speed, and to infer the placement of active replication origins (Figure 4A, B). Sequencing data was analysed using DNAscent software v3.0.2, an overhauled version of its predecessor, which originally detected BrdU incorporation into replicating DNA and identified fork direction and speed via the changing gradient of BrdU incorporation in the nascent strand (22). V3.0.2 now distinguishes between EdU and BrdU on the same molecule, thus more reliably identifying fork directionality and origins.

### Only a subset of potential origins are used as active replication origins during *P. Falciparum* schizogony

In contrast to ChIP-seq where aligned sequences and the corresponding sequence enrichments (i.e. peaks and summits) represent population level data, analysis with DNAscent v3.0.2 reveals snapshots of DNA replication dynamics on single molecules. We generated ∼2700× genome coverage for this analysis. Table 1 summarises the total number of left- and right-moving forks, origins and replication terminations detected. About two-thirds (2151 out of 3359) of the origins identified fired during the EdU pulse (Figure 4A, right replication schematic), while the rest fired before the pulse (Figure 4A, left replication schematic). Origins that fired during the EdU pulse were assigned a 1 bp coordinate, representing the centre of the EdU-labelled region, while origins that fired before the pulse—and may therefore have been anywhere between the two EdU-labelled regions—were assigned the entire region between the left- and right-moving forks. Mean G/C content in these identified origins was slightly higher (20.3%) compared to left- and right-moving forks (19.1–19.3%) which have G/C content similar to that of the whole genome (19.3%). Importantly, neither ORC1-ChIP sites nor active origin sites are necessarily fully and identically present in every single cell (however, where adjacent replication forks and origins appear on the same nanopore sequencing read, they must come from a single nucleus).

**Table 1.** DNAscent identifies origins, ongoing replication forks and terminations

			Length (bp)		G/C content (%)	
	Time	(*n*)	Mean	Median	Mean	Median
**Origins**	30 hpi	228	5843	1	20.16	15.48
	36 hpi	3131	4265	1	20.36	0.00
	All	3359	4372	1	20.34	0.00
**Left moving**	30 hpi	882	9199	8551	19.11	18.69
**forks**	36 hpi	13 021	8753	7991	19.28	18.68
	All	13 903	8781	8019	19.27	18.68
**Right moving**	30 hpi	867	9216	8675	19.08	18.64
**forks**	36 hpi	12 877	8798	8032	19.19	18.62
	All	13 744	8825	8068	19.18	18.62
**Sequenced**	30 hpi	497 590	32 150	28 331	19.56	18.82
**reads**	36 hpi	1 564 727	30 217	27 126	19.45	18.79
	All	2 062 317	30 684	27 386	19.47	18.80

Total number of origins, and left-/right-moving forks identified by DNAscent v3.0.2, and the total quality sequenced reads with the corresponding mean and median lengths and G/C content. Note that origins include both those that are mapped to a centre point (1 bp) and those derived from two separated left- and right-moving forks, in which case the origin is not mapped to a point, but to the entire inter-fork region.

This confirms that although ORC1 is widely and consistently distributed throughout the genome prior to and at the beginning of S-phase, only a few origins are active at mid-S-phase and even fewer at the beginning of S-phase. We identified over 4 times more active origins at mid S-phase (3131) than at early S-phase (228), suggesting that more potential origins are competent to fire at mid-S-phase (Table 1). Furthermore, there was clear evidence by mid S-phase for ‘efficient’ versus ‘inefficient’ origins, with some regions being detected as an active origin on many separate sequence reads, and others only once (Figure 4C).

### Overlap of active replication origins with ORC1 binding sites and other genomic features

**Figure 5.**
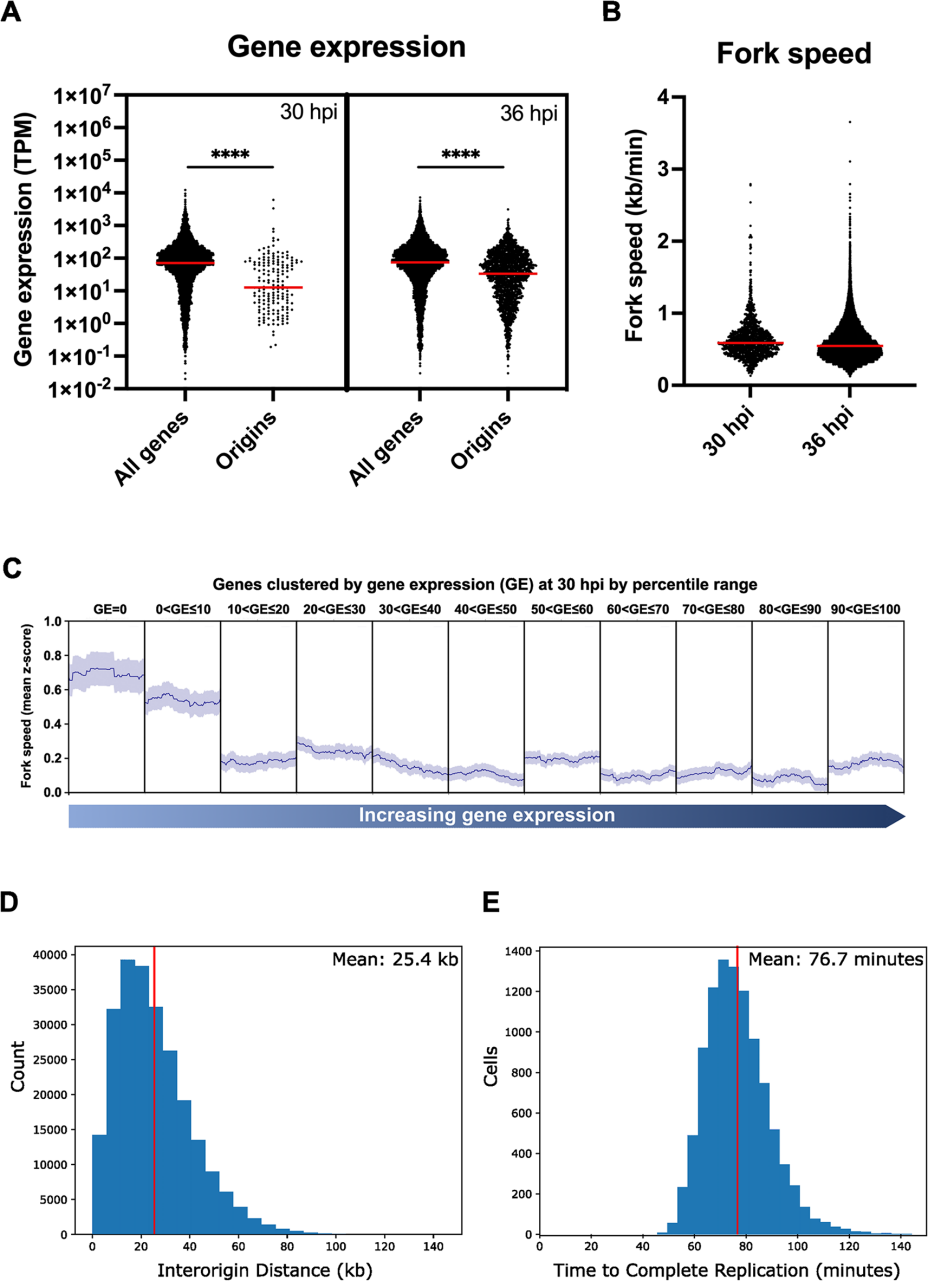
Median gene expression is lower in active origins, fork speed is faster in lowly transcribed regions, and Beacon calculus model shows good agreement with experimental data. (**A**) Gene expression (in transcripts per million, TPM) of genes that overlapped with active origins (±50 bp from the origin centre) at 30 and 36 hpi alongside expression of all genes at those time points. Red bars represent median gene expression. (**B**) Dot plot showing fork speed at 30 and 36 hpi, with the median represented by the red bar. (**C**) Mean fork speed *z*-score (dark blue line), representing the average deviation of the raw fork speed from the overall mean fork speed, mapped along coding sequences of genes clustered by gene expression at 30 hpi. The coding regions of genes within each cluster were scaled, with the left and right borders of the plot corresponding to the transcription start site and transcription end site, respectively. The standard error of the mean is shown as light blue shading above and below the mean fork speed. (**D**, **E**) Beacon calculus model of replication in non-telomeric regions of *P. falciparum* chromosome 1, found using the model shown in Supplementary Figure S6. Distributions of (D) inter-origin distances, and (E) total replication times are shown. The model shows good agreement with the distribution of inter-origin distances and fork speeds in DNA fibre-spreading data, as well as with the total replication time per nucleus. The model has a spatial distribution of 100 bp, and distributions are plotted from 10 000 simulations of chromosome 1 DNA replication. Vertical red lines show the mean of each distribution. Simulation of the model was done with the Beacon Calculus Simulator software v1.1.0.

We did not see any significant correlation between active origins and ORC1 ChIP sites—perhaps because active origins were 1–2 orders of magnitude less abundant than ORC1 binding sites. There was also no correlation between active origins and histone markers, predicted AP2 transcription factor binding sites, and chromatin accessibility. However, there was negative correlation between active origins and gene expression, as measured by previous transcriptomic studies conducted across the lifecycle (correlation coefficients of –0.1515 and –0.1736 at 30 and 36 hpi (Supplementary Table S9)). To examine transcriptional activity at these active origins, we calculated the median expression of genes with protein-coding sequences that overlap with origins that fired at 30 and 36 hpi and compared those against the median gene expression of all genes at the closest available time point in published data, i.e. 30 and 35 hpi (43). Median gene expression was significantly lower at active origin sites during both early and mid-S-phase (*P*-values: 9.88e–14 and 8.27e–43, respectively, Figure 5A). This suggests that origins are more likely to fire when positioned in genes that are low-expressing, and that this preference is even greater in origins fired early in schizogony.

GO term analysis of genes at the active origins revealed that most of the top 5 curated GO processes were related to the *var* gene family, which is largely transcriptionally silent during the replicative stages of the parasite (Supplementary Table S10). ORC1 has been suggested to have an important role in *var* gene regulation (60). Interestingly there was a positive correlation between ORC1 ChIP and origin activity at *var* genes (0.1298–0.3065), which differs from the lack of correlation across the whole genome (–0.0733 to –0.0014) (Supplementary Table S9).

Finally, building on the finding that origins fire preferentially in genes with low expression, we checked whether the progress of replication forks might be affected by transcription-replication conflicts. Indeed, the speed of forks was slower as they traversed the protein-coding sequences of most highly-expressed genes and faster in the lowest-expressed as well as transcriptionally silent genes at 30 hpi. Median fork speed was also significantly faster at the beginning than the middle of schizogony: 0.589 kb/min at 30 hpi versus 0.548 kb/min at 36 hpi (*P*-value = 3.24 e–08) (Figure 5B, C). The same trend was reported in previously published data from DNA fibre combing, although the absolute speeds measured by that method were markedly faster (1.388 kb/min in early trophozoites and 1.176 kb/min in mid/late trophozoites) (7). However, the fork speeds calculated here are much closer to those calculated in a more recent publication that utilised DNA spreading rather than combing (0.6–0.7 kb/min) (6).

### Modelling of replication origin activity in *P. Falciparum*

To check whether the single-molecule replication parameters measured here were consistent with the observed time periods taken to replicate a whole nucleus (5,6), we created a simple stochastic model of *P. falciparum* DNA replication in the Beacon Calculus (Supplementary Figure S6) (61). For simplicity, we used only *P. falciparum* chromosome 1, taking the median spacing between licensed origins on chromosome 1 as that measured by ORC1 ChIP (700 bp) and the average replication fork speed measured by DNAscent at 30 hpi (0.64 kb/min). Despite being deliberately simple, the model (Figure 5D, E) showed remarkably good agreement with the distribution of inter-origin distances and fork speeds measured by fibre spreading, as well as the total replication time per nucleus of 40–75 min, measured by McDonald and Merrick (6).

## DISCUSSION

This study represents the first empirical analysis of DNA replication origin activity in *Plasmodium*: a non-model organism and early-diverging eukaryote that replicates by the unique mode of schizogony. Besides this biological advance, it also represents a technological advance: the first application of powerful, newly evolved DNAscent software. This allowed us to map active origins on single DNA molecules by detecting two different nucleotide analogues in nascent DNA. A key finding was an unusual bias of origin firing towards areas of low gene transcription, suggesting an evolutionary pressure to avoid conflicts between transcription and origin-firing: a vulnerability that might be exploited with new antimalarial treatment strategies.

Firstly, we mapped ORC1 binding sites via ChIP-seq and found that *Plasmodium* ORC1 does not appear to organise the asynchronous replication of multiple nuclei within the same cell that is characteristic of schizogony. ORC1 entered the nucleus before S-phase started and remained there as the number of nuclei increased, being synthesised continuously, presumably to keep pace with the growing number of total genomes per cell. By contrast, proliferating cell nuclear antigen (PCNA) has a limited supply and shuttles between nuclei, providing a marker for actively replicating nuclei (5). There is also no clear evidence in our cell-level data for ORC1 degradation between rounds of replication, as occurs in some systems (although previous data suggest that ORC1 is degraded during the late schizont stage, after S-phase has finished (60)). In a cell that does not conform to once-and-only-once genome replication, it should be more efficient to keep ORC bound to each genome for the next round until S-phase is completed, presumably while adding newly-synthesised ORCs to each daughter genome. Alternatively, ORC may be evicted into the nucleoplasm at the end of each round, partitioned into daughter nuclei and then re-bound to DNA. In fact, if ORC was loaded onto DNA exclusively prior to S-phase and then progressively evicted after each of 4–5 replicative rounds, this might explain why ORC1 binding sites detected by ChIP were extremely abundant. However, it would then be unnecessary to produce additional ORC1 throughout S-phase, as more genomes are generated, arguing against this model and in favour of ORC1 loading for each new replicative round.

ChIP revealed that ORC binding sites are extremely abundant and not sequence-specific in *P. falciparum*. Nevertheless, clear ChIP peaks showed that binding is not totally random: there was a clear preference for G/C enrichment. This is unusual, since ORC in most species—including *S. cerevisiae* and *S. pombe*—prefers A/T-rich sequence (62). However, even a relative ‘enrichment’ in a genome of only ∼19% G/C remains low. It is unlikely to signify a preference for G-quadruplex DNA structures, as suggested in human cells (51,52). A basic G/C preference could explain why G-quadruplex-forming motifs were somewhat enriched in the ChIP peaks, but the density of peaks was far greater than the predicted density of quadruplex motifs in the *P. falciparum* genome (63,64). In fact, a simple G/C binding preference in ORC1 may be sufficient to generate clear sites of enrichment in what is a highly A/T-biased genome.

Surprisingly, no other features of the chromatin landscape seemed to determine ORC1 distribution. In other systems, ORC1 associates with intergenic regions (which was not the case here), with transcription start sites, acetylated histones, open chromatin, and CpG islands (which are near-absent in *P. falciparum* (65)). ORC1 showed no correlation with epigenetic marks and a negative correlation with transcription factor binding sites and accessible chromatin, although this could be confounded by their opposite bias towards A/T-richness. The exception was a positive correlation between ORC1 and HP1, a marker of heterochromatin: this persisted even outside the telomeres, where ORC1 may play separate, non-S-phase roles in maintaining telomere structure and silencing (48,60).

ORC1 was evidently bound to the genome very densely at G/C-enriched sites—although it may not be present at every site in every cell, since ChIP-seq is a population-level technique. Nevertheless, as in most eukaryotic cells, it seems that only a fraction of these sites were used as active origins. Activation did appear to be determined by the chromatin environment—more precisely, the transcriptional landscape. However, instead of a bias towards firing origins in highly transcribed genes (66), as in human cells, *P. falciparum* showed the opposite bias. An imperative to reduce transcription/replication conflicts by preferentially activating origins in low-expressed genes may drive this bias. The molecular mechanism remains unknown, but these cells are probably fundamentally replication-stressed (7) and limited in their ability to activate checkpoints for DNA damage repair, lacking recognisable homologues of PIKK checkpoint kinases and also lacking the non-homologous end-joining repair pathway (15). It could therefore be particularly important to evolve a system that avoids conflict between RNA polymerases and actively firing origins.

ORC1-ChIP yielded so many sites that there was no overall correlation between these sites and active origins identified via DNAscent. In *S. cerevisiae* (67), where a greater proportion of origin sites are actively used, a correlation can be detected, but in metazoans only 5–20% of ORC sites may be used (68). Nevertheless, some sites were used more efficiently than others, similar to the situation in yeast and other systems (62), because some active origins were detected in similar positions on many DNA strands, whereas others were not. What determines origin efficiency in *P. falciparum* is unknown. Lack of transcription may be entirely responsible, or there may be other factors besides those tested here. Interestingly, the large *var* gene family may present an extreme example of the preference for firing origins in transcriptionally inactive genes. Here, amongst ∼60 genes that are almost entirely silenced, ORC1 placement and origin activation correlated well, whereas in the rest of the genome, which is more variably transcribed, ORC1 sites were so dense versus the density of active origins that there was no overall correlation.

Consistent with the concept that *Plasmodium* has evolved to minimise transcription/replication-origin conflicts, we found that replication forks moved slowly through the most highly transcribed genes, suggesting that such conflicts do inhibit efficient DNA replication. Exacerbating this stressor—for example, by inhibiting DNA repair or replicative helicase activity—could be a route to generate unsurvivable replication stress in malaria parasites. In fact, replication may already be unusually slow and prone to stalling in these cells. The ability to measure replication fork velocity is another powerful aspect of the DNAscent software, and is orthogonal to the more traditional measurement of fork velocity via DNA combing. Fork velocities in *P. falciparum* DNA were strikingly lower when measured by DNAscent or by DNA fibre spreading (6) than they were by the usual ‘gold standard’ of DNA combing (7), which usually stretches DNA fibres at a reproducible 2 kb/μm (69). *P. falciparum* DNA must be alkali-treated prior to combing to remove the haemozoin that interferes with combing (7), and this may result in over-stretching and hence an overestimation of fork velocity. Conversely, both DNA spreading and DNAscent may give an underestimate because they rarely measure very long fibres and hence neglect the longest forks, whereas hundreds of kilobases can often be measured contiguously on combed DNA. A combination of both these issues could contribute to disparities in measured fork velocity: 1.0–1.4 kb/min as measured by combing, versus 0.6–0.7 kb/min by spreading or DNAscent. Assuming that the lower measurements are correct, they are strikingly slow compared to either human cells or yeast. High rates of fork asymmetry and stalling were measured in our previous DNA-combing study (7) (these are independent of absolute velocity), and they suggest that *P. falciparum* operates under considerable replication stress—possibly exacerbated by its A/T-rich and highly repetitive genome, which may cause DNA polymerases to move unusually slowly.

In addition to providing the first map of replication origin activity in *P. falciparum*, this study paves the way to investigate other interesting aspects of malaria parasite biology. Firstly, is origin specification similar in other *Plasmodium* genomes such as *P. knowlesi*, which are not as G/C-poor as *P. falciparum* (9)? Here, a simple G/C preference in ORC1 binding might not be sufficient to yield clear binding sites. Secondly, how do the demands of gametogenesis—where genome replication is extremely fast but there is little active transcription (70)—differ from those of erythrocytic schizogony? Gametogenesis may resemble an extreme version of replication in early *Xenopus* or *Drosophila* embryos, where origins fire every 10–20 kb (71). Indeed ORC1 binding sites may be extremely dense in this system simply because they are needed uniquely in male gametes, whereas they are never all activated in schizogony. It is evident that much remains to be studied about the very unusual cell cycles pursued by malaria parasites.

## DATA AVAILABILITY

Plasmid (pTK-BSD) and transgenic parasite line (*P. falciparum* 3D7 ORC1-3xHA + pTK-BSD) used in this study may be available upon request through the corresponding authors. ChIP-seq and DNAscent data have been deposited at GEO, under accession numbers GSE208757 (ChIP-seq) and GSE210010 (DNAscent). DNAscent v3.0.2, as well as DNAscent detect, DNAscent forkSense, and dnascent2bedgraph which are all part of DNAscent v3.0.2, are available under GPL-3.0 at https://github.com/MBoemo/DNAscent (also available on Zenodo at https://doi.org/10.5281/zenodo.7590289) and relevant Python scripts are available at https://github.com/FTotanes/DNAscent (also available on FigShare at https://doi.org/10.6084/m9.figshare.21982673.v2). Any additional information required to reanalyse the data reported in this paper is available from the corresponding authors upon request.

## Supplementary Material

gkad093_Supplemental_Files
