# Supplementary material for: A genome-wide map of DNA replication at single-molecule resolution in the malaria parasite Plasmodium falciparum
Source: Nucleic Acids Res. 2023 Feb 20;51(6):2709–24. doi: 10.1093/nar/gkad093 (PMC10085703; doi:10.1093/nar/gkad093)
Supplement: gkad093_Supplemental_Files [file gkad093_supplemental_files.zip › Supplementary_revised submission 2nd round.pdf]

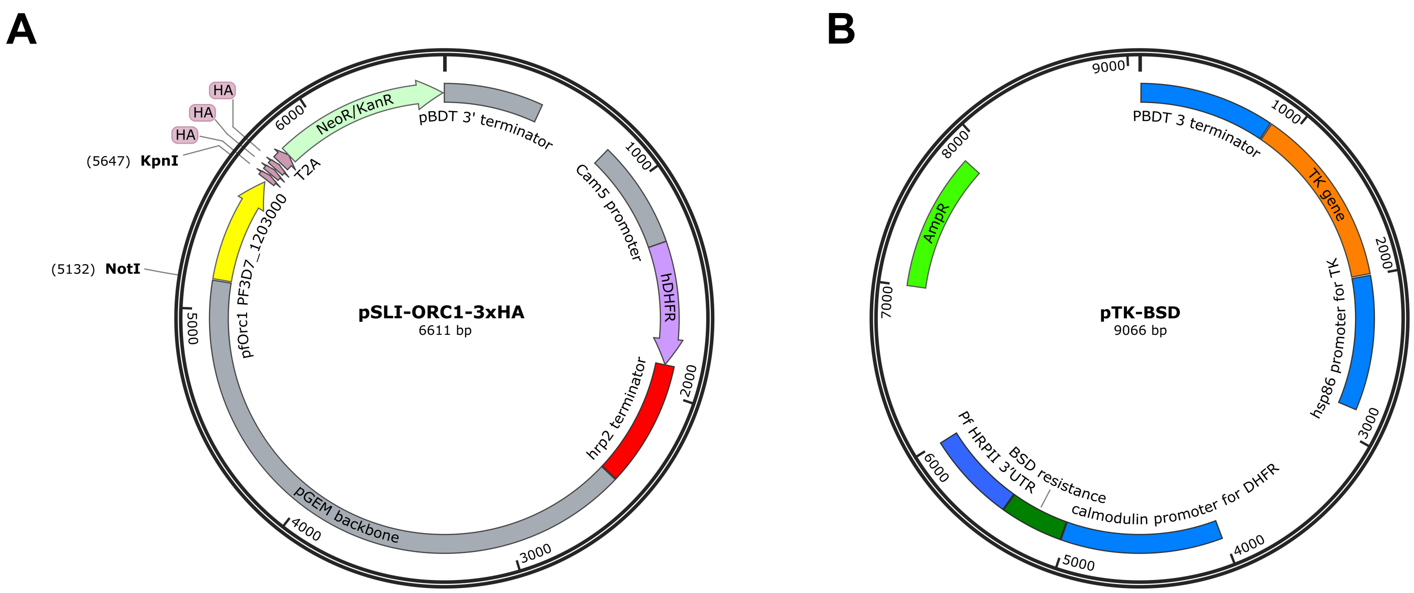

Supplementary Figure 1: Plasmids used in the generation of genetically modified parasites

A) Selection-linked integration plasmid used to C-terminally tag *P. falciparum* 3D7 with 3xHA.

B) Transfected plasmid used to allow parasites to episomally express thymidine kinase.

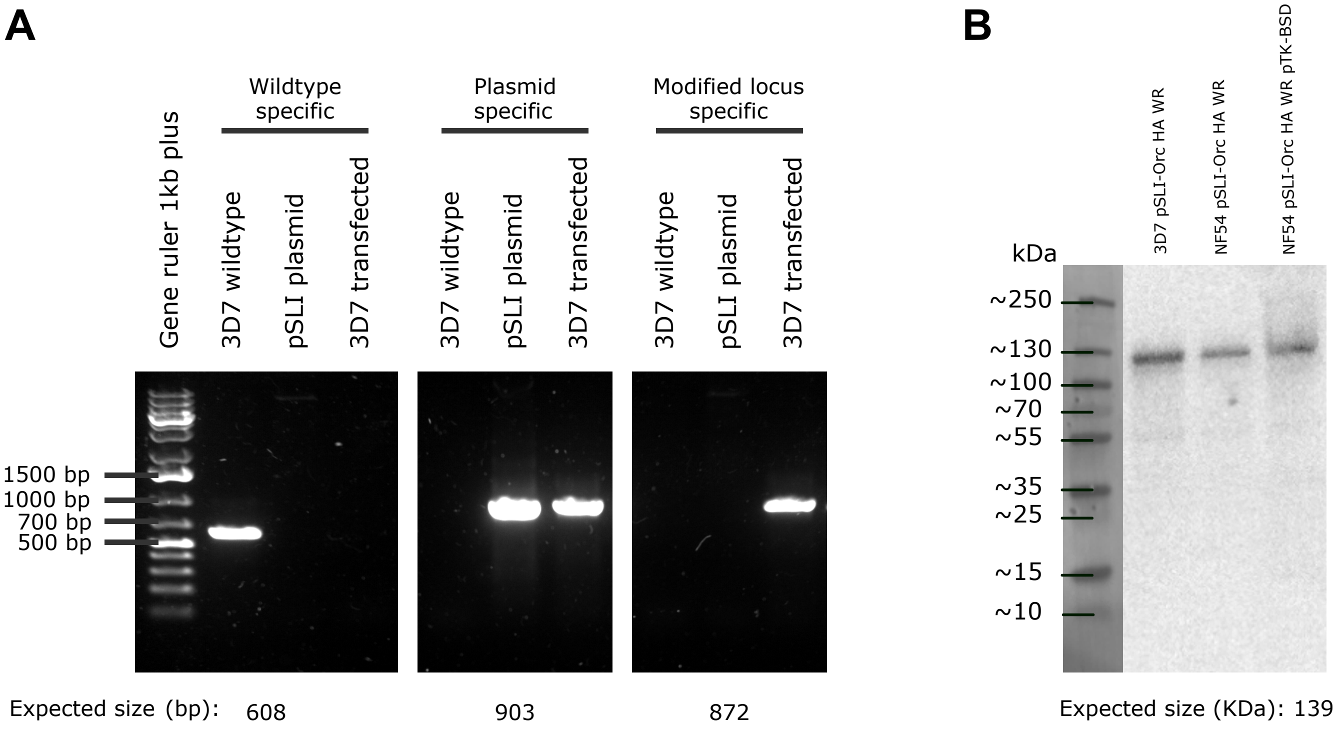

Supplementary Figure 2: Confirmation of transfection and gene tagging

A) PCR confirmation of successful integration of 3xHA and neomycin resistance gene into *orc1* (PF3D7_1203000). Primers used are listed in Supplementary Table 1.

B) Western blot confirmation of HA-tagging of ORC1.

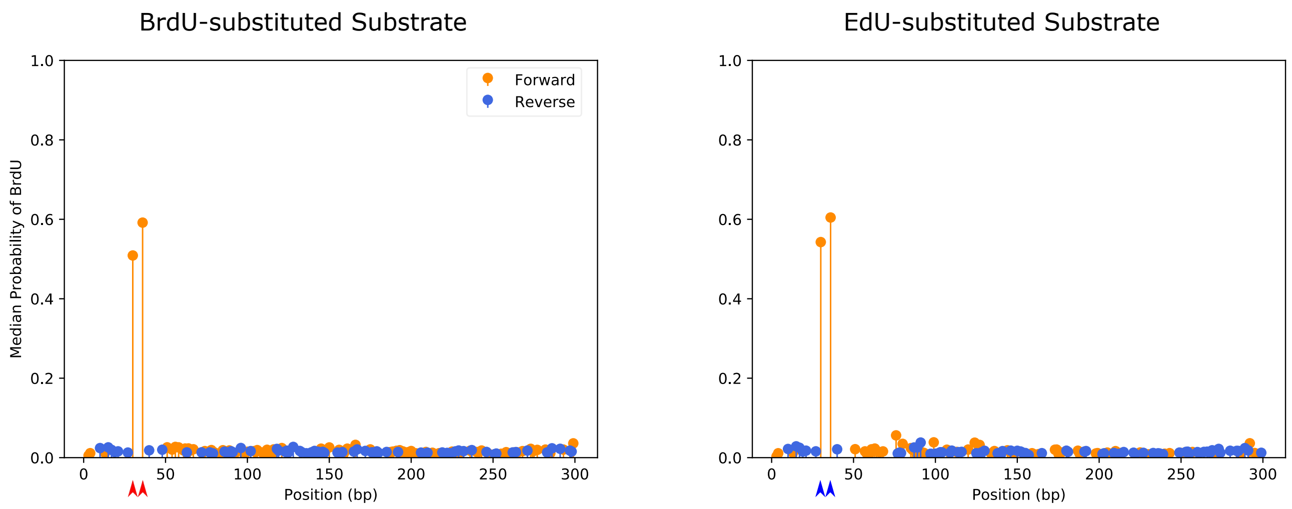

Supplementary Figure 3: Median probabilities of BrdU and EdU called using DNAscent v2.0.2

Median probabilities of BrdU called using DNAscent v2.0.2 on the forward (blue) and reverse (orange) strands of primer extension reads from Muller *et al.*, 2019 (22) where either BrdU or EdU were incorporated into two known positions (30 and 36 bp; red arrows for BrdU; blue arrows for EdU) on the forward strand. Reads analysed had a mapping length greater than or equal to 100 bp and mapping quality greater than or equal to 20 were used, resulting in N=453 reads for the BrdU-substituted substrate (left) and N=594 reads for the EdU-substituted substrate (right).

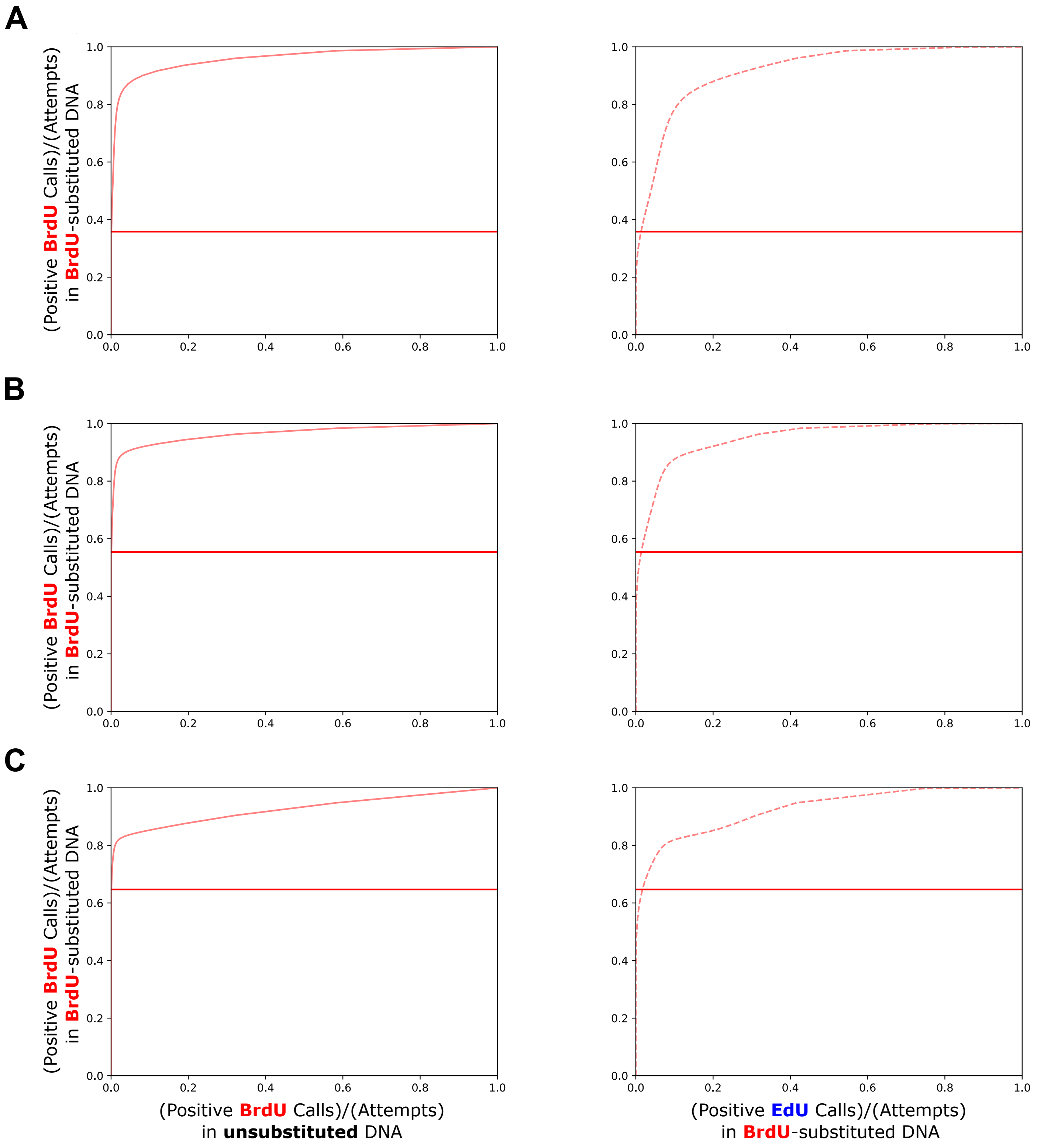

Supplementary Figure 4: Receiver operator characteristic curves for *S. cerevisiae* reads

Receiver operator characteristic (ROC) curves for *S. cerevisiae* reads from Muller *et al.*, 2019 (22) where BrdU substitution was measured by mass spectrometry to find (A) 26%, (B) 49%, and (C) 79% substitution. Curves show the number of positive analogue calls, defined as thymidine positions where DNAscent v3.0.2 assigned a probability of that analogue (BrdU or EdU) greater than a probability threshold, divided by number of thymidine positions. Points along the ROC curves indicate different probability thresholds above which an analogue call is considered positive. Horizontal lines intersect the curves at probability threshold of 0.5. Each curve was computed using N=1000 reads with a mapping quality of at least 20 and a mapping length of at least 1 kb to the *S. cerevisiae* sacCer3 assembly.

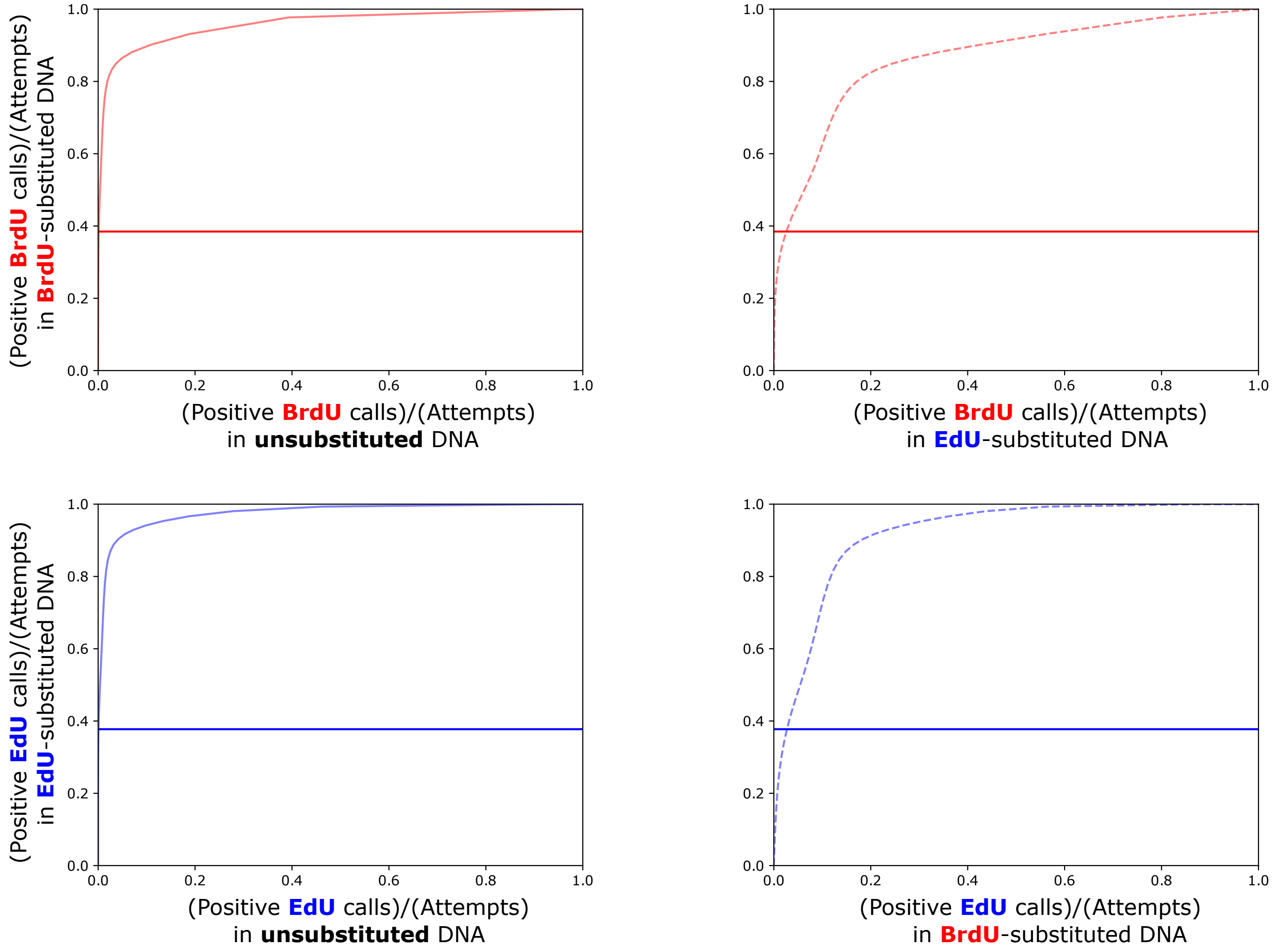

	Substrate		
	**EdU-substituted DNA**	**BrdU-substituted DNA**	**Unsubstituted DNA**
**EdU (positive calls)/attempts**	0.3774	0.0272	0.0009
**BrdU (positive calls)/attempts**	0.0260	0.3846	0.0014

Supplementary Figure 5: Receiver operator characteristic curves for *P. falciparum* reads

Receiver operator characteristic (ROC) curves for *P. falciparum* reads, where the probability of BrdU and EdU at each thymidine position was measured by DNAscent v3.0.2. The analogue-substituted reads for this benchmark were taken from a biological repeat of the sequencing run used to generate training data, and hence these reads are independent of the training dataset. As in the training dataset, EdU- and BrdU-treated *P. falciparum* reads were given distinct barcodes, sequenced on the Oxford Nanopore platform, and demultiplexed using Guppy. Reads were considered analogue-substituted if DNAscent v2.0.2 called made positive BrdU calls (probability of BrdU > 0.5) in at least 20% of thymidine positions across the read. Similar to Supplementary Figure 5, each curve was computed using 1000 reads with a mapping quality of at least 20 and a mapping length of at least 1 kb to the *P. falciparum* 3D7 ASM276v2 assembly and points along the ROC curves show probability thresholds above which an analogue call is considered positive. Horizontal lines intersect the curves at probability threshold of 0.5. The table shows the values on the x- and y-axis where the horizontal line intersects the ROC curve.

```
1  fast = 100000;
2  L = 6400; //chr1 length (100 bp resolution)
3  v = 6.4; //fork velocity (in 100-bp-per-minute)
4  fr = 0.001; //origin firing rate
5  spacing = 7.; //spacing between licensed origins (100 bp resolution)
6
7  //process definitions
8  Fp[i] = {chr![i],fast}.[i <= L] -> {~chr?[i+1],v}.Fp[i+1];
9  Fm[i] = {chr![i],fast}.[i >= 0] -> {~chr?[i-1],v}.Fm[i-1];
10 Ori[i] = {fire,fr}.(Fm[i] || Fp[i]) + {chr?[i],fast};
11 License[i] = [i < L] -> ( {licenseOri, fast/spacing}.( Ori[i] || License[i+1] )
12                         + {pass, fast}.License[i+1] );
13
14 //system
15 License[0];
```

Supplementary Figure 6: Source code of the Beacon Calculus model of the DNA replication of *P. falciparum*

Source code of the Beacon Calculus model of the DNA replication of *P. falciparum* chromosome 1 with 100-bp resolution. The model includes parameters for the length of *P. falciparum* chromosome 1 (Line 2), a uniform rate of fork movement (Line 3), a uniform firing rate for all origins (Line 4), and an average spacing between licensed origins (Line 5). The fork processes (Lines 8-9) and the origin process (Line 10) behave as in Boemo, 2020 (60). An origin licensing process (Lines 11-12) distributes licensed origins across the chromosome with an average spacing of 700 bp.

Primer name	Target	Details	Sequence	Length	Fragment size
GT_orc1_gene_FW	WT Genomic DNA	Genome specific	CAAATTTGTCGTAAAGCTTTCG	22	608
GT_orc1_3'UTR_RV		Genome specific	ATGCAAACAACAATATGGGG	20	
GT_orc1_gene_FW	Modified locus	Genome specific	Same as GT_orc_gene_FW	22	872
GT_NeoR_RV		Plasmid specific	TCGGTCTTGACAAAAAGAACC	21	
GT_pGEMbackbone_FW	Plasmid	Plasmid specific	CAATTTCACACAGGAAACAGC	21	903
GT_NeoR_RV		Plasmid specific	Same as GT_NeoR_RV	21	

Supplementary Table 1: Genotyping primers

Genotyping and control primer sequences used to confirm successful integration of 3xHA and neomycin resistance gene into *orc1* (PF3D7_1203000) and expected fragment sizes.

24 hpi ORC1 ChIP inter-summit distances (bp)

Chromosome	(n)	mean	median	min	max	10th percentile	25th percentile	75th percentile	90th percentile	Chromosome length
Pf3D7_01_v3	334	1786	716	96	20002	303	440	2177	4441	640851
Pf3D7_02_v3	516	1818	831	74	21263	297	445	2520	4488	947102
Pf3D7_03_v3	623	1680	892	144	15009	313	445	2187	4218	1067971
Pf3D7_04_v3	699	1695	727	119	29594	296	433	2131	4314	1200490
Pf3D7_05_v3	738	1787	906	86	18671	316	476	2465	4424	1343557
Pf3D7_06_v3	781	1805	774	62	38384	304	432	2243	4659	1418242
Pf3D7_07_v3	823	1737	842	54	20112	297	444	2275	4353	1445207
Pf3D7_08_v3	812	1676	749	32	17052	302	430	2166	4223	1472805
Pf3D7_09_v3	771	1964	960	77	26483	313	460	2693	4899	1541735
Pf3D7_10_v3	927	1810	853	53	32133	322	483	2539	4385	1687656
Pf3D7_11_v3	1017	1988	846	76	35943	318	462	2743	4890	2038340
Pf3D7_12_v3	1256	1789	867	73	21192	316	466	2248	4591	2271494
Pf3D7_13_v3	1464	1976	963	11	24713	310	471	2630	4772	2925236
Pf3D7_14_v3	1645	2000	1003	52	23541	319	477	2784	4994	3291936
Overall	12406	1848	863	11	38384	310	457	2471	4640	23292622

30 hpi ORC1 ChIP inter-summit distances (bp)

Chromosome	(n)	mean	median	min	max	10th percentile	25th percentile	75th percentile	90th percentile	Chromosome length
Pf3D7_01_v3	271	2252	884	39	20212	283	438	3046	5362	640851
Pf3D7_02_v3	393	2387	1120	8	22037	284	490	3424	5714	947102
Pf3D7_03_v3	450	2333	1144	64	23039	343	562	3167	5678	1067971
Pf3D7_04_v3	540	2200	832	58	30820	297	463	3070	5539	1200490
Pf3D7_05_v3	530	2488	1287	61	26484	362	547	3337	5849	1343557
Pf3D7_06_v3	549	2567	1112	111	39147	346	512	3276	6643	1418242
Pf3D7_07_v3	585	2451	1199	44	21136	313	512	3433	6119	1445207
Pf3D7_08_v3	569	2394	948	70	21905	315	483	3206	6548	1472805
Pf3D7_09_v3	525	2885	1629	125	21792	365	622	4042	7061	1541735
Pf3D7_10_v3	694	2419	1121	11	32303	316	506	3345	6076	1687656
Pf3D7_11_v3	721	2789	1143	57	36000	321	516	3837	6764	2038340
Pf3D7_12_v3	952	2376	1009	84	34858	314	478	3225	6034	2271494
Pf3D7_13_v3	1033	2800	1369	5	33700	333	530	3882	6788	2925236
Pf3D7_14_v3	1171	2809	1359	37	23799	330	509	3987	6998	3291936
Overall	8983	2556	1156	5	39147	323	509	3490	6430	23292622

Supplementary Table 2: ORC1 ChIP-seq inter-summit distances

Descriptive statistics of calculated distances between summits called using MACS2 on ORC1 ChIP-seq at 24 and 30 hpi per chromosome.

24 hpi summits					
Motif	Consensus sequence	Length	Weighted G/C content	Enrichment	p-value
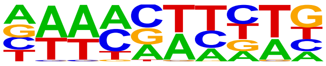	NAAMVWYBTG	10	32.02%	1.33	1.00E-20
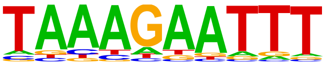	TAAAGAATTT	10	18.47%	1.14	1.00E-17
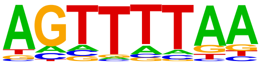	AGTTTTAA	8	23.35%	1.11	1.00E-16
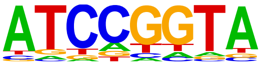	ATCCGGTA	8	45.55%	1.14	1.00E-16
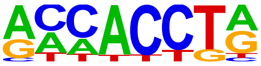	AMCACCTR	8	47.39%	1.23	1.00E-15
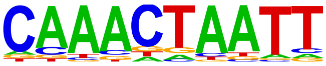	CAAACTAATT	10	26.94%	1.21	1.00E-14
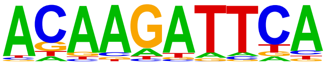	ACAAGATTCA	10	30.22%	1.23	1.00E-12
**30 hpi summits**					
Motif	Consensus sequence	length	Weighted G/C content	Enrichment	p-value
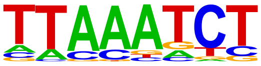	TTAAATCT	8	25.31%	1.14	1.00E-21
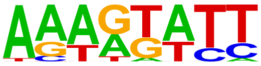	AAARTATT	8	21.58%	1.12	1.00E-16
**24 hpi peaks**					
Motif	Consensus sequence	length	Weighted G/C content	Enrichment	p-value
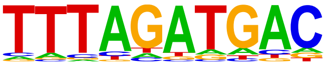	TTTAGATGAC	10	32.23%	1.26	1.00E-13
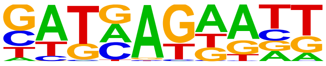	GATVAGAATT	10	37.32%	1.53	1.00E-12
**30 hpi peaks**					
Motif	Consensus sequence	length	Weighted G/C content	Enrichment	p-value
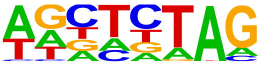	ARCTCTAG	8	34.96%	1.14	1.00E-12

Supplementary Table 6: *De novo* Motifs
Significant motif structure and consensus sequences identified in ORC1 ChIP summit ± 50 bp using HOMER motif analysis software v4.11.1 with their corresponding weighted GC-content, enrichment scores and p-values. Motifs with an enrichment p-value ≤ 1 e-12 were considered significant.

		(n) sequences	total length	G4 count	Enrichment
**ORC1 ChIP peaks**	24 hpi	8843	4599930	101	2.05
	30 hpi	6785	3337581	101	2.82
**ORC1 ChIP summits ± 50 bp**	24 hpi	12420	1242000	8	0.60
	30 hpi	8997	899700	13	1.35
**whole genome**		16	23332831	249	

Supplementary Table 7: Enrichment of G-quadruplex forming motifs in ORC1 ChIP data

G4Hunter was used (with a stringent threshold of 1.7 to identify high-confidence motifs) to identify G4 forming motifs in ORC1 ChIP peak and summit sequences at 24 and 30 hpi, and in the whole genome. Enrichment was calculated as the ratio of the total number of G4 per bp in the peak or summit sequences and the total number of G4 per bp in the whole genome.

		ORC1 ChIP	
		24 hpi	30 hpi
AP2		-0.2078	-0.1918
ATACseq	5 hpi	-0.3085	-0.2965
	10 hpi	-0.5751	-0.5653
	15 hpi	-0.4839	-0.4734
	20 hpi	-0.6302	-0.6138
	25 hpi	-0.5514	-0.5343
	30 hpi	-0.6207	-0.5990
	35 hpi	-0.4919	-0.4714
	40 hpi	-0.5764	-0.5537
H2A	40 hpi	0.0338	0.0293
H2A.z	10 hpi	-0.0309	-0.0335
	20 hpi	0.0069	0.0021
	30 hpi	0.0004	-0.0066
	40 hpi	-0.1139	-0.1141
H3K4me3	10 hpi	0.0009	-0.0005
	20 hpi	0.0093	0.0049
	30 hpi	-0.0001	-0.0030
	40 hpi	-0.0331	-0.0314
H3K9ac	10 hpi	-0.0007	-0.0031
	20 hpi	0.0016	-0.0035
	30 hpi	-0.0780	-0.0812
	40 hpi	-0.1493	-0.1528
HP1	rings	0.4434	0.4663
	trophs	0.3402	0.3699
	schizonts	0.5485	0.5737
HP1 (no telomeres)	rings	0.3764	0.3836
	trophs	0.2555	0.2704
	schizonts	0.4993	0.5095

Supplementary Table 8: Spearman correlation between ORC1 ChIP data and known epigenetic markers

Genome-wide correlation coefficients between ORC1 ChIP log2ratios at 24 and 30 hpi and log2ratios of histone markers, chromatin accessibility, as well as HP1 placement at different timepoints in the parasite lifecycle were calculated using deeptools multiBigwigSummary (bin size of 100 bp). Correlation coefficients between ORC1 ChIP and HP1 outside telomeric regions were also calculated. Negative correlations are highlighted in red and positive correlations in blue.

		DNAscent origins log2ratio		
		30 hpi	36 hpi	All
DNAscent origins log2ratio	30	1.0000	0.0435	0.4243
	36	0.0435	1.0000	0.9033
	All	0.4243	0.9033	1.0000
ORC1 ChIP log2ratio (whole genome)	24	-0.0014	-0.0534	-0.0597
	30	-0.0035	-0.0699	-0.0733
ORC1 ChIP log2ratio (var genes)	24	0.1298	0.2403	0.2461
	30	0.1501	0.2933	0.3065
AP2	AP2	-0.0138	-0.0048	-0.0099
ATACseq R1	5	0.0139	0.0088	0.0111
	10	0.0037	0.0062	0.0066
	15	0.0064	-0.0027	-0.0015
	20	-0.0020	-0.0027	-0.0027
	25	-0.0134	-0.0197	-0.0217
	30	-0.0200	-0.0415	-0.0413
	35	-0.0207	-0.0628	-0.0617
	40	-0.0029	-0.0285	-0.0267
ATACseq R2	5	0.0086	-0.0043	-0.0025
	10	0.0141	0.0067	0.0092
	15	0.0030	-0.0005	0.0005
	20	-0.0052	-0.0054	-0.0070
	25	-0.0213	-0.0345	-0.0375
	30	-0.0304	-0.0557	-0.0576
	35	-0.0263	-0.0715	-0.0714
	40	-0.0049	-0.0431	-0.0407

		DNAscent origins log2ratio		
		30 hpi	36 hpi	All
H2A	40	-0.0002	-0.0025	-0.0027
H2A.z	10	0.0069	0.0111	0.0135
	20	0.0041	-0.0014	0.0005
	30	0.0160	0.0134	0.0186
	40	0.0185	0.0449	0.0490
H3K4me3	10	0.0039	-0.0072	-0.0034
	20	0.0012	-0.0059	-0.0044
	30	0.0003	0.0002	0.0025
	40	-0.0030	-0.0020	-0.0001
H3K9ac	10	0.0039	-0.0040	-0.0012
	20	0.0000	-0.0114	-0.0098
	30	0.0138	0.0084	0.0139
	40	0.0197	0.0121	0.0207
HP1	rings	0.0074	0.0234	0.0226
	schizonts	0.0014	0.0158	0.0130
	trophs	0.0129	0.0288	0.0312
RNAseq	5	-0.0423	-0.0773	-0.0923
	10	-0.0614	-0.0857	-0.1046
	15	-0.0747	-0.0949	-0.1145
	20	-0.0815	-0.1118	-0.1297
	25	-0.0797	-0.1324	-0.1459
	30	-0.0734	-0.1512	-0.1622
	35	-0.0577	-0.1662	-0.1736
	40	-0.0284	-0.1376	-0.1420

Supplementary Table 9: Spearman correlation between DNAscent active origins and known epigenetic markers

Genome-wide correlation coefficients between DNAscent origin log2ratios (at 30 hpi, 36 hpi and both time points combined; over the whole genome or only at var genes) and log2ratios of histone markers, chromatin accessibility, HP1 placement, and gene expression (‘RNA-seq’) at different timepoints in the parasite lifecycle were calculated using deeptools multiBigwigSummary (bin size of 100 bp). Negative correlations are highlighted in red and positive correlations in blue.
